# The severely injured older patient: identifying patients at high risk for mortality using the Dutch National Trauma Registry

**DOI:** 10.1007/s00068-024-02738-x

**Published:** 2025-01-24

**Authors:** Sara van Ameijden, Mariska de Jongh, Martijn Poeze

**Affiliations:** 1 Network Emergency Care Brabant, Tilburg, The Netherlands; 2 Department of Surgery, Elisabeth-TweeSteden Hospital, Tilburg, The Netherlands; 3 Tilburg School of Social and Behavioral Sciences, Tilburg University, Tilburg, The Netherlands; 4 Department of Surgery, Division of Trauma Surgery, Maastricht University Medical Centre, Maastricht, The Netherlands

**Keywords:** Aged, Multi-trauma, Mortality, Risk factors

## Abstract

**Purpose:**

The incidence of severely injured older trauma patients is increasing globally, portraying high mortality rates. Exploring the demographics and clinical outcomes of this subgroup is essential to further improve specialised care at the right place. This study was performed to identify severely injured older patients at high risk for mortality by examining their characteristics and identifying prognostic factors contributing to mortality.

**Methods:**

A retrospective cohort study was conducted using data from the Dutch National Trauma Registry to identify all trauma patients aged 70 years and older from 2016 to 2022. Subgroup analyses for characteristics and outcomes were performed based on Injury Severity Score (ISS) 16–24 and ISS ≥ 25, as well as age groups of 70–79, 80–89 and ≥ 90 years. A logistic, backwards regression analysis was performed to identify predictors for mortality within each ISS groups.

**Results:**

In total, 10,901 patients were included. The mean age was comparable between the ISS groups (80.48 ± 6.8 vs. 80.54 ± 6.6 years). The main trauma mechanisms in both the ISS 16–24 and ISS ≥ 25 were low energy falls and bicycle accidents. The head and thorax were the most frequently injured body regions, with a significantly higher proportion of severe head injuries in the ISS ≥ 25 group (32.6% vs. 73.4%). Mortality rates increased significantly with higher injury severity (13.9% vs. 48.9%) and advancing age (22.6% vs. 32.4% vs. 35.8%). The most significant predictors of mortality in the ISS 16–24 group were an increase in ASA score and a GCS 3–8 at arrival (OR for GCS: 7.2 (95% CI 5.7–9.1), AUC 0.76). Similarly, in the ISS ≥ 25 group, an increased ASA score and a GCS 3–8 at arrival were the most significant predictors of mortality as well (OR for GCS: 10.8 (9.1–12.9), AUC 0.79). Although increasing age was also associated with a higher risk of mortality in both ISS groups, its impact was less significant than the aforementioned variables.

**Conclusion:**

Severe injuries in older patients are predominantly caused by low energy falls and bicycle accidents, leading to high mortality rates. A low GCS at arrival and high ASA scores are most strongly associated with an increased risk for mortality. Notably, despite the prevalence of severe injuries among the oldest patients, the proportion of intensive care unit admissions decreases markedly with age. This raises the question what feasible care for these often frail patients should comprise of and where this care should be provided, especially for those with severe pre-existent comorbidities.

**Level of evidence and study type:**

Level III, prognostic/epidemiological.

## Background

Severe trauma among the older population is an increasing global challenge. As populations age, older trauma patients are constituting a growing percentage of all trauma cases [1–3]. Currently, approximately 20% of the Dutch population is aged 65 years or older, with this proportion expected to rise significantly in the coming decades [4].

These older individuals are experiencing increased mobility and engaging in more leisure-time physical activities [5, 6]. Consequently, the percentage of older individuals within the trauma population is rising [7]. Between 2009 and 2015, patients aged 60 years and older accounted for 47.8% of the total trauma population in the Netherlands [8], with incidence rates increasing over the last decade [9]. Older trauma patients most commonly present themselves following minor falls and traffic accidents, resulting most often in traumatic brain injury (TBI), spinal injuries, and rib fractures [1, 3, 10–13]. The care of these patients imposes a significant burden on healthcare costs in the Netherlands, with a mean cost of €12,190 up to 2 years post-injury [14].

The older severely injured patients are at higher risk for mortality when compared to their younger counterparts. This increased risk is related to factors such as the high prevalence of comorbidities, polypharmacy, and limited physiological reserves. Additionally, and similar with the general trauma population, higher trauma severity, a low Glasgow Coma Scale (GCS) score, hypotension at admission, and respiratory complications also contribute to higher mortality rates among older patients [10, 11, 15–21]. However, during the past 15 to 20 years, multiple studies have revealed an overall decrease in mortality among older patients with severe injuries [2, 11, 13]. It is hypothesised that this trend is due to improvements in resuscitation, the development of interdisciplinary geriatric trauma care, the evolution of specialised trauma teams, and advancements in diagnostic modalities [2, 13, 22].

Given the ongoing increase in the yearly incidence of older patients with severe injuries, this study aims to provide an up-to-date overview of patient characteristics, clinical outcomes, and healthcare utilisation (e.g., length of hospital stay, intensive care admission) of these patients in the Netherlands. By distinguishing between the characteristics and outcomes of severely and very severely injured patients, as well as between older and very old patients, this study aims to offer a detailed and comprehensive analysis. Furthermore, this study aims to identify prognostic factors associated with a higher risk of mortality following both severe and very severe injury, with the goal of improving early identification of high-risk older patients. We hypothesise that age alone is a limited predictor of mortality and that the predictive value of prognostic factors differs between severely and very severely injured older patients.

## Methods

### Study design and data source

This nationwide descriptive and prognostic study utilised retrospective data extracted from the Dutch National Trauma Registry (DNTR). The DNTR includes data on all patients in the Netherlands who were admitted to the hospital through the emergency department (ED) within 48 h after trauma. Patients without vital signs upon arrival at the ED are not included in the DNTR database. Inclusion in the DNTR does not require patient consent, but patients have the option to opt out. Inclusion of the data requested for this study was approved under study number LTR23.03, and the data were provided to the authors in an anonymised format.

The DNTR contains a variety of information, including demographics, injury mechanisms, anatomical injury characteristics coded according to the Abbreviated Injury Scale (AIS) (version 2005, update 2008), injury severity as measured by the Injury Severity Scale (ISS), vital signs on admission, and various outcome variables such as mortality, length of stay, and functional outcomes [23]. The ISS score is calculated as a derivative of the AIS score, defined as the sum of the squares of the highest AIS grade in each of the three most severely injured body regions [24].

### Study population

This study included all patients in the DNTR database from 2016 to 2022 who were aged 70 years and older at the time of initial presentation and were classified as severely injured. While the older population is generally considered to include those aged 65 years and older [25], it has been suggested that mortality, when adjusted for the ISS score, increases from the age of 70 years. Therefore, 70 years was chosen as the age cut-off to define a patient as ‘elderly’ or ‘geriatric’ [26]. Age was stratified into cohorts of 10 years: 70–79, 80–89, and ≥ 90 years. Severe injury was defined as an ISS score of ≥16 [27], with the ISS scores further stratified into ‘severely injured’ (ISS score of 16–24) and ‘very severely injured’ (ISS score of ≥ 25). The reported American Society of Anesthesiologists (ASA) score reflects the patient’s pre-injury condition.

### Injury characteristics and mechanism

The injury characteristics for each body region were classified according to the AIS score. Severe injury (AIS score of ≥ 4) for each body region (head, neck, spine, thorax, abdomen, upper extremities, lower extremities, and external) was reported.

Regarding injury mechanisms, the study differentiated between traffic incidents (pedestrian, bicycle, motorised vehicle, other), low-energy falls (<3 times body height), high-energy falls (≥3 times body height), and other mechanisms (gunshot and knife injuries, blunt object injuries, burn injuries, and other mechanisms not covered by the aforementioned categories).

### Outcome measures

Hospital mortality was analysed as the primary outcome. The secondary outcomes were 30-day mortality, functional outcomes, length of hospital stay, and intensive care unit (ICU) admission. Functional outcomes were reported using the Glasgow Outcome Scale (GOS) [28].

### Statistical analysis

All statistical analyses were performed using IBM SPSS Statistics version 24.0 and R version 3.6.0 (2019-04-26). Nominal and categorical data are presented as count and percentage, while continuous data are presented as mean and standard deviation or median and interquartile range (IQR), depending on the distribution. Dichotomous and categorical variables were tested using the Pearson chi-square test (or the ordinal chi-square test if required) or Fisher’s exact test. Continuous variables were tested using the independent t-test, the Mann–Whitney U-test, or the Kruskal–Wallis H-test, depending on the distribution. All p-values of < 0.05 were considered statistically significant.

To identify predictors associated with increased mortality among severely and very severely injured older patients, the following variables were analysed using a stepwise, backward method: age; sex; number of body regions with an AIS score of ≥ 2; presence of severe injuries with an AIS score of ≥ 4 in the following body regions: the neck, spine, thorax, abdomen, external, upper extremities, and lower extremities; ASA classification; and GCS at arrival. Only complete weighted case analysis was performed. High inter-variable collinearity was observed between the GCS and severe head injury (AIS head score of ≥ 4); thus, the severe head injury variable was excluded from the regression model. The GCS was dichotomised into high (GCS of 9–15) and low (GCS of 3–8). Variables with a p-value of > 0.10 were removed from the model at each step [29].

The performance of the analyses was assessed based on goodness of fit, discrimination, and percentage of variability using the Hosmer–Lemeshow (H&L) test, area under the receiver operating characteristic curve (AUC), and Nagelkerke R^2^ value, respectively. An AUC of < 0.5 indicates no discrimination, 0.5 to 0.7 is considered acceptable, 0.8 to 0.9 is excellent, and > 0.9 is outstanding [30]. Additionally, calibration curves were plotted to demonstrate the agreement between predicted and observed mortality probabilities.

## Results

### Patient characteristics

**Fig. 1:**
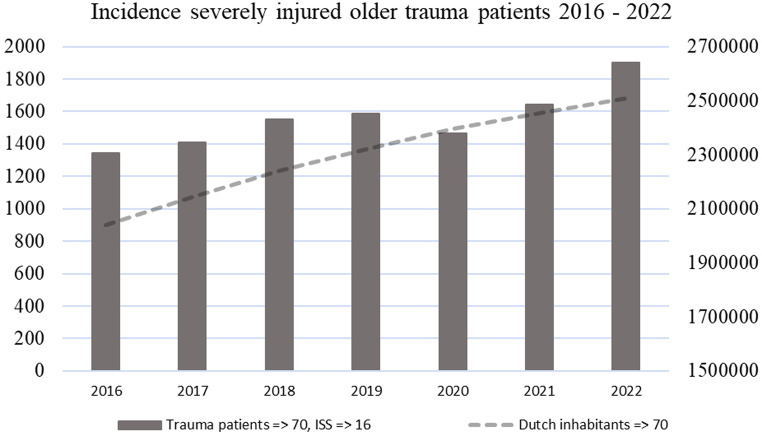
Yearly incidence of severely injured older trauma patients, 2016–2022

As shown in Table 1, a total of 10,899 patients aged 70 years and older were included in the cohort, with a yearly increase in incidence, as illustrated in Fig. 1 (the dotted trendline represents the number of Dutch inhabitants aged 70 years and older during the corresponding years [31]). The ISS 16–24 group comprised 6,572 patients, while the ISS ≥ 25 group comprised 4,327 patients. The mean age was comparable between the ISS groups, although the 80–89 year age group was slightly overrepresented in the higher ISS group (41.4% vs. 38.9%, *p* = 0.01). Both ISS groups had a higher proportion of men, with an increased percentage in the higher ISS group (56.1% men vs. 43.9% women, *p* < 0.001).

The most common causes of severe trauma in the ISS 16–24 and ISS ≥ 25 groups were low-energy falls (49.3% and 47.2%, respectively) and bicycle accidents (19.2% and 19.3%, respectively). Pedestrian accidents were more prevalent in the higher ISS group (3.3% vs. 2.5%, *p* < 0.001). In the ISS 16–24 and ISS ≥ 25 groups, the body regions most frequently sustaining severe injury (AIS score of ≥ 4) were the head (32.6% vs. 73.4%, *p* < 0.001), thorax (10.5% vs. 12.9%, *p* < 0.001), and spine (5.2% vs. 6.9%, *p* < 0.001). Patients in the higher ISS group had significantly lower GCS scores upon arrival and were more frequently admitted to a major trauma centre (67.6% vs. 51.7%, *p* < 0.001).

**Table 1:** Patient characteristics

*n* = 10,899	ISS 16-24 *n* = 6,572	ISS 25-75 *n* = 4,327	*p*-value
**Age**			
Mean (SD)	80.5 (6.8)	80.5 (6.6)	*p*=0.70
70-79 years	3347 (50.9%)	2147 (49.6%)	*p*=0.01
80-89 years	2555 (38.9%)	1791 (41.4%)	
90 years and above	670 (10.2%)	389 (9.0%)	
**Sex** (n, %)			*p*<0.001
Male	3390 (51.6%)	2429 (56.1%)	
Female	3181 (48.4%)	1897 (43.9%)	
**Injury mechanism** (n, %)			*p*<0.001
Traffic accident			
Bicycle	1232 (19.2%)	817 (19.3%)	
Motorised	488 (7.6%)	300 (7.1%)	
Pedestrian	161 (2.5%)	138 (3.3%)	
Other	44 (0.7%)	36 (0.9%)	
Low energy fall	3164 (49.3%)	1996 (47.2%)	
High energy fall	769 (12.0%)	543 (12.9%)	
Other	259 (4.0%)	259 (6.1%)	
Missing	295	136	
**Severe injury (AIS ≥ 4) (n, %)**			
Head	2140 (32.6%)	3178 (73.4%)	*p* < 0.001
Spine	339 (5.2%)	297 (6.9%)	*p* < 0.001
Abdomen	100 (1.5%)	177 (4.1%)	*p* < 0.001
Thorax	692 (10.5%)	558 (12.9%)	*p* < 0.001
Upper extremities	4 (0.1%)	1 (0.1%)	*p* = 0.368
Lower extremities	239 (3.6%)	206 (4.8%)	*p* = 0.004
External AIS	73 (1.1%)	179 (4.1%)	*p* < 0.001
**ASA** (n, valid %)			*p* < 0.001
ASA I	546 (9.2%)	303 (7.9%)	
ASA II	3132 (53.0%)	1924 (50.3%)	
ASA III	2069 (35.0%)	1463 (38.2%)	
ASA IV	151 (2.6%)	119 (3.1%)	
ASA V	6 (0.1%)	16 (0.4%)	
Missing	668	502	
**GCS** (n, valid %)			*p* < 0.001
3	293 (5.0%)	961 (24.8%)	
4-5	36 (0.6%)	161 (4.1%)	
6-8	132 (2.2%)	318 (8.2%)	
9-12	437 (7.5%)	489 (12.6%)	
13-15	4978 (84.7%)	2880 (50.3%)	
Missing	696	448	
**Type of trauma centre at initial presentation** (n, %)			*p*<0.001
MTC	3397 (51.7%)	2924 (67.6%)	
nMTC	3175 (48.3%)	1403 (32.4%)	
**Transfer from ED to a different hospital** (n, %)	323 (4.9%)	249 (5.8%)	*p*<0.001

All p-values were analysed using the chi-square test. Abbreviations: nMTC = non major trauma centre

### Mortality and recovery related to injury severity

Patients with an ISS of 25–75 had significantly higher hospital mortality (48.9% vs. 13.9%, *p* < 0.001) and 30-day mortality (57.7% vs. 19.6%, *p* < 0.001) than those with lower ISS scores, as shown in Table 2. Patients in the higher ISS group were more frequently admitted to the ICU (52.3% vs. 36.1%, *p* < 0.001) and had a significantly longer hospital stay (median [IQR] of 10 [13] vs. 8 [10] days, *p* < 0.001). In terms of functional outcomes, the lower ISS group showed significantly more instances of good recovery (16.5% vs. 10.4%), while the higher ISS group had a greater proportion of severe disability (31.3% vs. 17.9%).

**Table 2:** Outcomes related to injury severity

	ISS 16–24 *n* = 6,572	ISS 25–75 *n* = 4,327	*p*-value
**Hospital mortality** (n, %)			*p* < 0.001^#^
Non survivors	914 (13.9%)	2114 (48.9%)	
Survivors	5658 (86.1%)	2212 (51.1%)	
Missing	0	1	
**30-day mortality** (n, %)			*p* < 0.001^#^
Non survivors	1137 (19.6%)	2320 (57.7%)	
Survivors	4484 (77.2%)	1584 (39.4%)	
Missing (n)	765	306	
GOS* (n, %)			*p* < 0.001^#^
Good recovery	868 (16.5%)	214 (10.4%)	
Moderate disability	2914 (55.3%)	914 (44.6%)	
Severe disability	944 (17.9%)	641 (31.3%)	
Persistent vegetative state	36 (0.7%)	45 (2.2%)	
Missing	506 (9.6%)	236 (11.4%)	
LOS (days)*			
(median-IQR)	8 (IQR 10)	10 (IQR 13)	*p* < 0.001^¥^
**ICU admission %**	36.1%	52.3%	*p* < 0.001^#^

* Excluding deceased patients, # Chi-square test, ¥ Mann-Whitney test

Abbreviations: GOS = Glasgow Outcome Scale, LOS = Length of hospital stay, ICU = Intensive Care Unit

### Mortality and recovery related to age

As shown in Table 3, in-hospital mortality significantly increased with each advancing decade of age (22.6% vs. 32.4% vs. 35.8%, *p* < 0.001). A similar trend was observed in 30-day mortality (27.7% vs. 41.3% vs. 48.6%, *p* < 0.001). As age increased, the proportion of ICU admissions significantly decreased (52.2% vs. 36.4% vs. 17.6%, *p* < 0.001). The length of hospital stay was 1 day shorter in the oldest age group than in the younger groups, with a median [IQR] of 8 [9] days for patients aged ≥ 90 years compared with 9 [12] days for those aged 70–79 years and 9 [10] days for those aged 80–89 years (*p* < 0.001). In terms of functional outcomes, the distribution across outcome categories was comparable between the age groups; septuagenarians showed a slightly higher incidence of severe disability, while octogenarians exhibited more moderate disability.

**Table 3:** Outcomes related to age

	70-79 years *n* = 5,493	80-89 years *n* = 4,346	≥90 years *n* = 1,059	*p*-value
**ISS ≥ 25**	39.1%	41.2%	36.7%	
**Hospital mortality** (n, %)				*p*<0.001^#*^
Non survivors	1241 (22.6%)	140 (32.4%)	379 (35.8%)	
Survivors	4252 (77.4%)	2938 (67.6%)	680 (64.2%)	
Missing	1	0	0	
**30-day mortality** (n, %)				*p*<0.001^#*^
Non survivors	1366 (27.7%)	1625 (41.3%)	466 (48.6%)	
Survivors	3444 (69.8%)	2178 (55.3%)	466 (46.6%)	
Missing (n)	561	409	101	
GOS* (n, %)				*p*<0.001^#^
Good recovery	608 (15.3%)	376 (13.8%)	98 (15.8%)	
Moderate disability	2059 (51.8%)	1460 (53.7%)	309 (49.9%)	
Severe disability	904 (22.7%)	558 (20.5%)	123 (19.9%)	
Persistent vegetative state	37 (0.9%)	31 (1.2%)	12 (1.9%)	
Missing	370 (9.3%)	296 (10.8%)	77 (12.3%)	
LOS (days)*				
(median-IQR)	9 (IQR 12)	9 (IQR 10)	8 (IQR 9)	*p* = 0.02^¥^
**ICU admission %**	52.2%	36.4%	17.6%	*p*<0.001^#^

* Excluding deceased patients, #* Chi-square test for trend, # Chi-square test, ¥ Mann-Whitney test

Abbreviations: GOS = Glasgow Outcome Scale, LOS = Length of hospital stay, ICU = Intensive Care Unit

### Identifying predictors related to hospital mortality

**Fig. 2:**
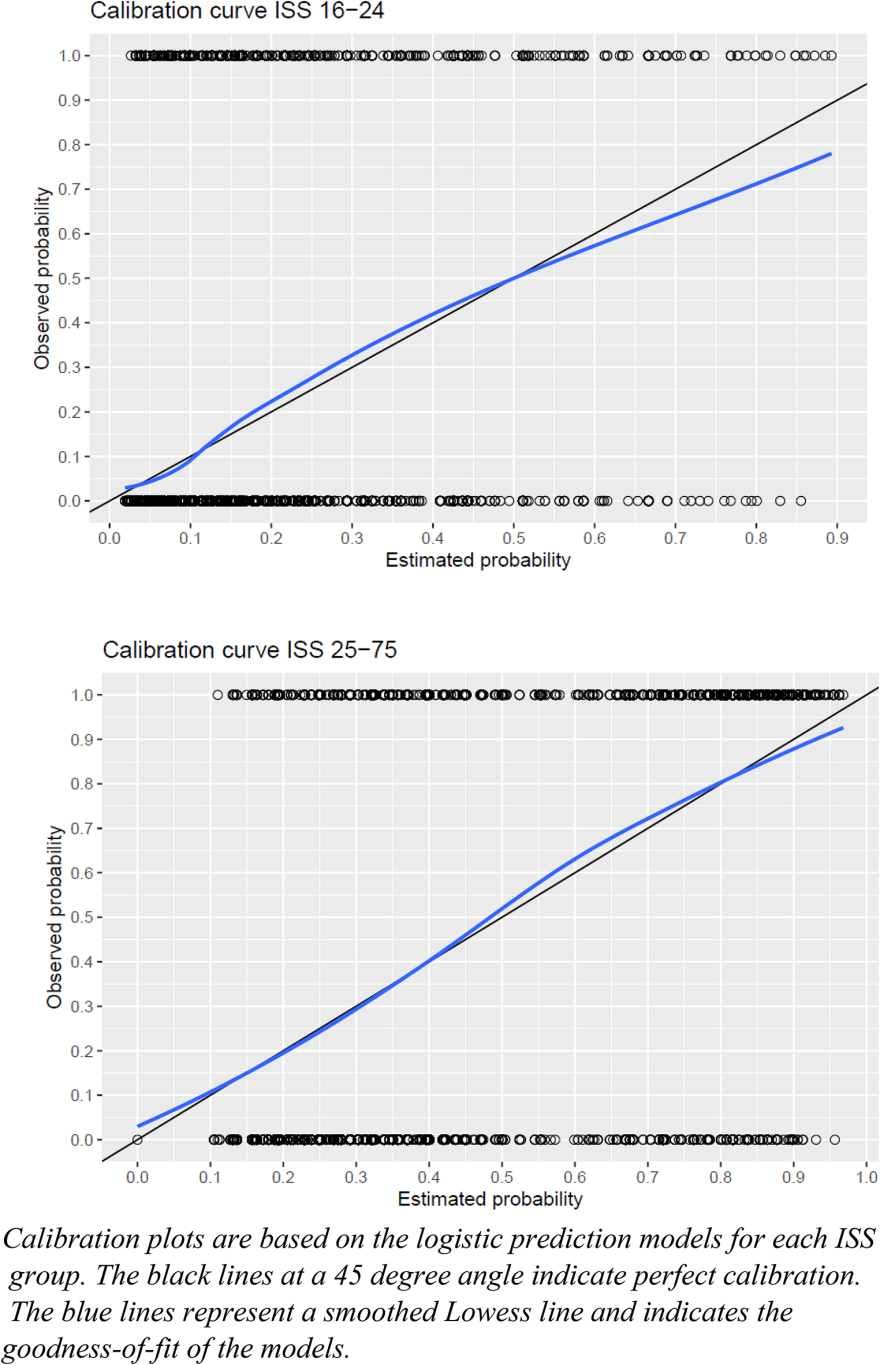
Calibration curves for predicted versus observed mortality ISS 16–24 and ISS 25–75

For patients with an ISS of 16–24, multiple regression analysis with backward selection identified the following variables as predictors of hospital mortality: age, sex, number of injuries with an AIS score of ≥ 4, ASA classification, low GCS score at arrival, AIS thorax score of ≥ 4, and AIS external score of ≥ 4. The H&L test result was 0.170, indicating an adequate fit. The AUC was 0.76 (95% CI, 0.74–0.78), and Nagelkerke’s R^2^ was 0.19. The calibration curve, shown in Fig. 2, indicates that for an ISS of 16–24, the model tended to overestimate the predicted mortality rate as the observed probability increased.

For patients with an ISS of 25–75, the predictors of hospital mortality were age, sex, ASA classification, low GCS score at arrival, and AIS spine score of ≥ 4. The fit of this model was higher than that of the model for the lower ISS group, with an H&L test result of 0.37, an AUC of 0.79 (95% CI, 0.78–0.81), and Nagelkerke’s R^2^ of 0.33. The calibration curve for the ISS 25–75 model showed less overestimation of predicted mortality, closely following the ideal line (See Fig. 2).

**Table 4:** Backward logistic regression analysis of mortality for ISS 16–24 and ISS 25–75

	ISS 16-24			ISS 25-75		
**Factor**	**OR**	**p-value**	**95% CI**	**OR**	**p-value**	**95% CI**
**Age (years** **)**						
70-79	***ref***			***ref***		
80-89	2.52	<0.001	2.07-3.05	1.98	<0.001	1.67-2.36
90+	4.18	<0.001	3.19-5.47	3.02	<0.001	2.25-4.06
**Sex (female)**	0.71	<0.001	0.60-0.84	0.81	0.009	0.69-0.95
**Number of AIS-coded injuries**						
1	***ref***			***ref***		
2	1.37	0.162	0.88-2.14	0.81	0.200	0.68-1.12
3	1.13	0.588	0.72-1.78	0.77	0.130	0.55-1.08
≥4	1.71	0.007	1.16-2.54	1.22	0.149	0.93-1.60
**Comorbidities**						
ASA 1	***ref***			***ref***		
ASA 2	1.23	0.332	0.82-1.83	1.28	0.133	0.93-1.76
ASA 3	2.34	<0.001	1.57-3.51	1.77	<0.001	1.27-2.47
ASA 4	6.55	<0.001	3.86-11.11	5.17	<0.001	2.99-8.92
ASA 5	7.12	0.042	1.07-47.30	4.43	0.08	0.84-23.33
**GCS 3-8**	7.22	<0.001	5.71-9.13	10.84	<0.001	9.08-12.94
**AIS thorax ≥ 4**	1.74	<0.001	1.33-2.27	0.80	0.085	0.62-1.03
AIS external ≥ 4*	4.0	<0.001	2.62-8.81	-		-
AIS spine ≥ 4**	-		-	1.88	<0.001	1.38-2.56

* Variable not included in the last regression block for ISS 25–75, removed in the last step considering *p* ≥ 0.10

** Variable not included in the last regression block for ISS 16–24, removed in the last step considering *p* ≥ 0.10

Only complete case analysis was performed. For ISS 16–24, 5,314 cases were included. For ISS ≥ 25, 3,420 cases were included

## Discussion

This study demonstrated an increasing incidence of severely injured trauma patients aged 70 years and older in the Netherlands from 2016 to 2022. A high hospital mortality rate was observed, particularly among those with an ISS score of ≥ 25 and aged 90 years and older. For both severely and very severely injured older patients, a low GCS at arrival and high ASA classification emerged as the most significant predictors of mortality, while the predictive value of injuries to specific body regions varied between the ISS groups.

In our cohort, severe injury was most commonly caused by low-energy falls and bicycle accidents. Previous research consistently shows that low-energy falls are often the leading mechanism of injuries among severely injured older patients [1, 2, 13, 16, 32] and usually occur after a fall from a standing height [11]. Given that the Netherlands has one of the highest levels of cycling worldwide [33], it is unsurprising that bicycle injuries are a common cause of severe trauma among older patients. Bicycle injuries in this population often occur while mounting or dismounting the bicycle and frequently result in fractures and intracranial injuries [34].

Remarkably, the distribution of trauma mechanisms was similar between severely and very severely injured older patients. One might expect a higher proportion of high-energy mechanisms (e.g., high-energy falls, motor vehicle accidents) among the very severely injured. However, sustaining severe trauma even after relatively mild incidents is common in the older trauma population and has been noted as a contributing factor to the undertriage of older trauma patients [35]. Although current Dutch guidelines recommend that at least 90% of all trauma patients with an ISS score of ≥ 16 should be taken directly to the nearest major trauma centre (MTC) [36], our study also highlighted the challenge of undertriage. Only 51.7% of severely injured and 67.6% of very severely injured older patients initially presented at an MTC. The identification of high-risk older trauma patients is hindered by the poor sensitivity of current triage criteria for this population [35]. Therefore, the development of geriatric specific triage tools should be considered more attentively, especially since current geriatric specific triage criteria are very variable, focus on different physiological thresholds, and lack consensus on the optimal tool [37–40]. Considering that a combination of multiple less severe injuries (i.e., AIS ≤ 3) can still result in an ISS ≥ 16, further research is needed to assess the impact of different injury combinations on mortality and outcomes across various levels of trauma centres. This may imply that the combination of (minor) injuries to multiple body regions should be considered when triaging older trauma patients to an MTC or non-MTC.

An important question that arises when discussing undertriage is the role of intensive trauma care and initial presentation at a MTC for different groups of severely injured older patients, particularly given the ongoing debate about whether MTC care improves survival and functional outcomes in this population [41–43]. This study did not examine potential differences between patients who initially presented at a MTC versus a non-MTC. It is possible that these groups differ intrinsically, and these differences could influence pre-hospital decisions to transport patients to an MTC or non-MTC, as well as in-hospital decisions regarding ICU admission. For example, our study showed that ICU admission rates decreased with age, despite the fact that injury severity was comparable across age groups. Despite evidence that ICU admission benefits mortality even in the oldest patients, ICU rejection rates increase with age [44]. With advancing age, patients are more frequently deemed ‘non-eligible’ for ICU admission by healthcare professionals because of comorbidities, frailty, and low pre-existing functional status [45]. Additionally, the role of advanced care planning and goals of care in the Netherlands in providing appropriate care needs to be considered. Approximately 10% of older patients referred to the emergency department have documented preferences regarding ICU admission, with this proportion increasing with age [46]. This may partly explain the decrease in ICU admissions with advancing age and influence healthcare professionals’ assessments of whether severely injured older patients would benefit from more intensive trauma care.

TBI was the most prevalent severe injury in this study, affecting 73% of patients with an ISS ≥ 25. This was also reflected in the GCS scores at arrival, with patients in the ISS ≥ 25 group having significantly lower GCS scores than those in the ISS 16–24 group. Older patients are at higher risk of substantial TBI and adverse outcomes [18, 47] due to factors such as decreased brain elasticity, increased brain atrophy, and the use of anticoagulants [48–50]. In this study, TBI and subsequent low GCS scores were identified as significant predictors of unfavourable outcomes, particularly mortality. A low GCS score at arrival was the most impactful prognostic factor for hospital mortality, associated with a 7- to 11-times higher risk of death in severely and very severely injured older trauma patients, respectively.

As mentioned earlier, severe injuries resulting from bicycle accidents were common among older trauma patients in this study. The relationship between bicycle accidents and subsequent TBI as a contributor to mortality is important to consider. This relationship has also been observed in other national Dutch data registries, where 59% of cyclists who died following bicycle accidents in 2019 were aged 70 years and older [51]. With more than 50% of cyclists aged 65 years and older using e-bikes [52], the growing popularity of e-bikes has contributed to severe trauma among older patients. Poos et al. [53] reported that e-bikers sustain multiple severe injuries twice as often as conventional cyclists and are more likely to suffer severe TBI. Given the high prevalence of severe TBI following bicycle accidents and the strong association between severe TBI and mortality, it is crucial for trauma teams to be particularly vigilant when older patients present after bicycle accidents. Additionally, the Dutch government should consider implementing primary prevention measures, such as mandatory helmet use or preventive programs for safe cycling among the elderly [54]. Furthermore, early and thorough radiological assessment should be conducted to improve clinical outcomes [55].

In addition to a low GCS, severe thoracic injury was also associated with higher mortality. Severe thoracic trauma was prevalent in our cohort; age-related reductions in bone quality increase the risk of rib fractures [18], which are more common than injuries to internal thoracic organs among older patients [56]. Respiratory complications, such as pneumonia, occur in approximately 10% of older patients with rib fractures [57], contributing to increased mortality. Although severe thoracic trauma was more common in the higher ISS group, it was not a significant predictor of hospital mortality. This suggests that the higher rate of ICU admissions in the higher ISS group may help reduce the impact of aformentioned complications. This finding highlights the importance of early diagnosis, adequate pain management, respiratory physiotherapy, and mobilisation in reducing adverse events following severe thoracic trauma.

Although the overall prevalence of severe external injuries was low, an injury classified as AIS external with a severity score of ≥ 4 was identified as a risk factor for mortality in the ISS 16–24 group. External injuries encompass a variety of conditions, including skin injuries, asphyxia, hypothermia, and burn injuries, with burn injuries being the most common cause of a high external AIS score [58]. Therefore, older patients presenting with these types of injuries should be considered high-risk and considered for early intensive care.

Hospital mortality increased with both age and trauma severity. Both advancing age and a high ASA classification were significant independent prognostic factors for hospital mortality, particularly in the ISS 16–24 group. Previous studies have also identified comorbidities as significant predictors of mortality [16, 17, 19, 59], whereas the role of age becomes less significant as age increases [17, 19, 60]. It is important to note that the studies mentioned above typically used specific comorbidities (e.g., cardiovascular disease, renal comorbidities) as predictors, whereas the ASA classification in this study may serve as a more generalised (and potentially weaker) factor in relation to age. However, as hypothesised, a high ASA classification still had a stronger predictive value for mortality than age alone in both ISS groups. Therefore, high age alone does not define whether a patient is at high risk, and the role of age in assessing the risk of severely injured older patients warrants further consideration. Additional patient-specific characteristics, such as pre-existing frailty, comorbidities, and correlated polypharmacy, should be comprehensively evaluated to accurately determine whether an older patient is at high risk for mortality and would benefit from increased care intensity. More detailed patient file analysis is needed in future research to better understand the association between these factors and mortality, which could ultimately lead to new insights into feasible trauma care for severely injured older patients.

Among surviving patients, most experienced moderate or severe disability, regardless of age. Physical limitations, reduced independence, and diminished social networks are recognised as contributors to a decreased quality of life after trauma [61]. The increased disability observed in older surviving trauma patients underscores the significant burden that severe trauma imposes on a population that often has pre-existing frailty, and highlights the importance of pursuing geriatric rehabilitation after hospital discharge for patients who experience functional loss during admission. However, several barriers must be overcome to promote this rehabilitation pathway, such as the active involvement of patients and/or caregivers in decision-making, the use of in-hospital triage tools to identify suitable care for geriatric patients, and the quality of discharge information [62].

### Study limitations

This study is limited by its retrospective design; missing data could not be accounted for in the database used. In the DNTR, the functional outcome is described using the GOS score, but the GOS is only validated for assessing disability and social participation after TBI [63]. However, the DNTR provides a GOS score for patients without neurological brain injuries as well. This, combined with inter-observer bias, should be considered when interpreting the results. Additionally, comorbidities in patients are only measured using the ASA classification. The use of the ASA classification as a proxy for comorbidities could be refined in future studies to further improve the identification of high-risk patients, or the ASA classification could be replaced with more specific indicators (e.g., frailty scores or the Charlson Comorbidity Index). Other potentially significant predictors, such as medication use, pre-existing level of functioning, or frailty, are not available in the DNTR and therefore could not be reported. Furthermore, the generalisability of the results is limited by the use of national data.

Regarding the prediction models, this study is limited by the relatively low Nagelkerke R^2^ values, indicating that a substantial part of the variance cannot be explained by these models, particularly in the ISS 16–24 model. This could be partially improved by assessing the predictive value of specific comorbidities rather than using the ASA classification. The ISS 16–24 model also overestimated predicted mortality, making the model less reliable for triaging high-risk patients or assessing quality of care parameters for this group.

## Conclusion

Severe trauma among older patients leads to high mortality rates, especially as age increases, and is mainly caused by low-energy falls and bicycle accidents. Older trauma patients often present with severe head trauma, and given that a low GCS score at arrival is a significant predictor of mortality, there is a critical need for primary prevention and heightened vigilance from trauma teams when managing these patients. Additionally, age and pre-existing comorbidities are identified as prognostic factors for hospital mortality and should be considered to improve early identification and intensive care monitoring of high-risk older trauma patients. Future research should further explore the role of comorbidities, polypharmacy, and advanced care planning preferences in the care of the often frail, severely injured older trauma patient to determine where feasible care should be delivered.

## Data Availability

No datasets were generated during the current study. Data requests of the Dutch National Trauma Registry can be submitted to the Landelijk Netwerk Acute Zorg (LNAZ).
